# Extracellular matrix and fibroblast injection produces pterygium-like lesion in rabbits

**DOI:** 10.1186/s40659-018-0165-8

**Published:** 2018-06-05

**Authors:** Judith Zavala, Julio C. Hernandez-Camarena, Brenda Salvador-Gálvez, José E. Pérez-Saucedo, Amin Vela-Martinez, Jorge E. Valdez-García

**Affiliations:** 1 Tecnologico de Monterrey, Escuela de Medicina y Ciencias de la Salud, Ave. Morones Prieto 3000, 64710 Monterrey, NL Mexico; 2 Hospital Universitario “Dr. José E. González”, Universidad Autónoma de Nuevo León, Madero y Dr. Aguirre Pequeño, C.P. 64460 Monterrey, NL Mexico

**Keywords:** Pterygium, Fibroblast, Rabbit, Extracellular matrix, Animal model

## Abstract

**Background:**

Translational research to develop pharmaceutical and surgical treatments for pterygium requires a reliable and easy to produce animal model. Extracellular matrix and fibroblast are important components of pterygium. The aim of this study was to analyze the effect of the subconjunctival injection of fibroblast cells (NIH3T3 cell line) and exogenous extracellular matrix in rabbits in producing a pterygium-like lesion.

**Methods:**

Six 3-month-old white New Zealand rabbits were injected with 20,000 NIH3T3 cells and 5 µL of Matrigel in the right conjunctiva, and with only 5 µL of Matrigel in the left conjunctiva. The eyes were photographed under a magnification of 16× using a 12-megapixel digital camera attached to the microscope on day 1, 3 and 7. Conjunctival vascularization was measured by analyzing images to measure red pixel saturation. Area of corneal and conjunctival fibrovascular tissue formation on the site of injection was assessed by analyzing the images on day 3 and 7 using area measurement software. Histopathologic characteristics were determined in the rabbit tissues and compared with a human primary pterygium.

**Results:**

The two treatments promoted growth of conjunctival fibrovascular tissue at day 7. The red pixel saturation and area of fibrovascular tissue developed was significantly higher in right eyes (p < 0.05). Tissues from both treatments showed neovascularization in lesser extent to that observed in human pterygium. Acanthosis, stromal inflammation, and edema were found in tissues of both treatments. No elastosis was found in either treatment.

**Conclusions:**

Matrigel alone or in combination with NIH3T3 cells injected into the rabbits’ conjunctiva can promote tissue growth with characteristics of human pterygium, including neovascularization, acanthosis, stromal inflammation, and edema. The combination of Matrigel with NIH3T3 cells seems to have an additive effect on the size and redness of the pterygium-like tissue developed.

## Background

Pterygium is a benign fibrovascular growth of the ocular surface commonly associated to discomfort and red eye, and as disease progresses is often related to decreased vision (topographic astigmatism) and ocular motility restriction in severe cases [1]. A wide variety of pro-inflammatory cytokines induced by fibrogenic growth factors, oxidative stress and DNA methylation have been implicated in the pathogenesis of pterygium. Since some of these factors are affected by exposure to ultraviolet (UV) light, current evidence from multiple sources suggests that individuals with high exposure to sunlight are at increased risk of pterygium [2]. Despite the extensive research, there is no clear understanding of the pathogenesis of pterygium, but one of the most important factors contributing to the pathogenesis are the neoplastic changes of limbal stem cells associated to UV light exposure and the possible role of oncogenic virus (Human papillomavirus) [3].

Epidemiological studies report that the prevalence of pterygium in Latino population is elevated (16% overall), especially in people with low income and low educational status, increased age and with men having a higher prevalence compared to female (2:1) [4]. The definite treatment is often surgical excision with the use of adjuvant antiproliferative drugs (mitomycin C, 5-fluorouracil, doxycycline or bevacizumab), which still need standardization and, in the best scenario, still report variable recurrence rates (0.1–33.3%) [5–7]. In vitro models focused on the pathogenesis of pterygium to explore the intercellular signaling pathways in epithelial cells and fibroblasts show promising results [8, 9], but animal models to support the evidence are needed. To date, three animal models for pterygium have been described, using injection of human epithelial pterygium cells, exogenous extracellular matrix or UV scattered radiation in rabbit and mice [3, 10, 11]. The results of the rabbit model using UV scattered light are focused on the computational prediction of the size and shape of the tissue growth, with no histological analysis. The mouse model showed histological characteristics of human pterygium, however, manipulation of rabbits for ophthalmological procedures is easier given that the ocular structure and size resembles more to that of human.

Hence, the purpose of this study was to develop an animal model for pterygium using subconjunctival injection of murine fibroblast cells (NIH 3T3 cell line) and exogenous extracellular matrix (Matrigel) in white New Zealand rabbits suitable for its implementation on both basic pharmacologic/molecular research on pterygium pathogenesis and on novel surgical techniques.

## Methods

All procedures were conducted according to the Guide for the Care and Use of Laboratory Animals and were approved by Institutional Animal Care and Use Committee. A total of six 3-month-old white New Zealand rabbits were used. Animals were anesthetized with intramuscular injection of ketamine HCl (30 mg/kg) and xylazine (5 mg/kg) and topical anesthesia with tetracaine hydrochloride (2 eye drops). Rabbits received subconjunctival injection of 20,000 murine fibroblast cells (NIH 3T3 cell line) and 5 µL of Matrigel (Corning Inc., New York, US) on the perilimbal temporal bulbar conjunctiva of the right eye using a 1 ml 30G needle. Left eyes were injected with 5 µL of Matrigel under the perilimbal temporal bulbar conjunctiva (control). The eyes were photographed under a magnification of 16× using a 12-megapixel digital camera attached to the microscope (World Precision Instruments, Inc. FL, US) on day 1, 3 and 7. Conjunctival vascularization was measured on day 1, 3 and 7 by analyzing images using Adobe Photoshop CS5 (Version 12.0, Adobe, San Jose, CA) color histograms to measure red pixel saturation on a 6 × 6 mm area on the site of injection. Area of corneal and conjunctival fibrovascular tissue formation on the site of injection and surrounding area was assessed by analyzing the images on day 3 and 7 using area measurement software (Adobe Photoshop CS5).

After 7 days, surgical specimens were obtained under anesthesia and were immediately fixed in 10% buffered formalin (pH = 7.3) for at least 8 h, routine histopathologic process was followed and tissue was embedded in paraffin. Immediately after obtaining conjunctival specimens, all rabbits were euthanized (day 7) using a lethal dose (200 mg/kg) of intravenously-administered pentobarbital (Penta-Hypnol^®^, Agrovet MArket, Lima, Peru). Each sample was serially cut into at least 10 four micron-thick sections, mounted and stained with hematoxylin/eosin staining method. All specimens were evaluated by the same pathologist to limit subjective variation during the observation and counting process. Histopathologic characteristics including neovascularization, epithelial inflammation, stromal inflammation, acanthosis, elastosis and edema were determined in the rabbit tissues and compared with a human primary pterygium.

Histological changes were semi-quantitatively classified into six groups. Vascular density was defined as the average vessel count in six high power fields (HPF, 400×) in the areas appearing as the most vascularized foci. The whole pterygium specimen was examined and 6 HPF which seemed to have the greatest vascular density were selected; all vessels lined by endothelium were considered, given that the presence of endothelium makes it possible to differentiate a vessel from the pseudo-vascular canal. Epithelial and stromal inflammation were graded with the following scale: grade 0, absent or sparse inflammatory cells in the tissue; and grade 1, significant patchy or diffuse inflammatory cells infiltration. Acanthosis was graded based on the number of epithelial cells found (N) in the unaltered epithelium, using the following scale: grade 0, absent or any number smaller than N; grade 1, N – 1½N; and grade 2, 1½N – 2 N. The percentage of elastosis and edema were described as the proportion of elastotic changes and edema, respectively, to the whole fibro connective stroma and graded with the following scale: grade 0, absent; grade 1, element found on less than 50% of the tissue; and grade 2, element found on more than 50% of the tissue. The sections were analyzed using standard light microscopy (Carl Zeiss, Axiostar plus, Germany) under 400× and 40× magnification for vascular density, epithelial and stromal inflammation, and acanthosis; and elastosis and edema, respectively. Statistical analysis was performed using paired t-test and analysis of variance (ANOVA) for mean comparisons, statistical significance was considered with a p value < 0.05.

## Results

**Fig. 1:**
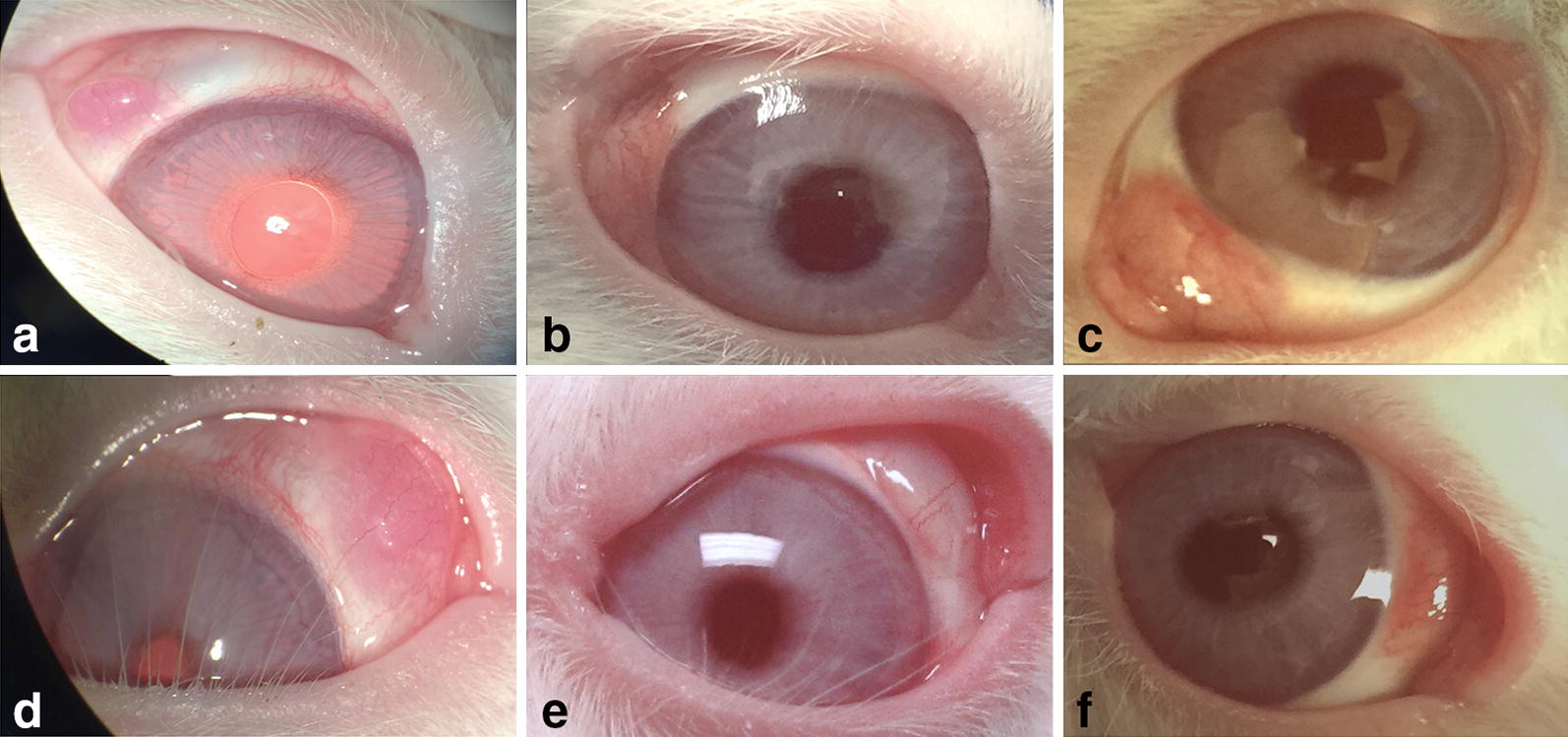
Right eye of rabbit 4 at 1, 3 and 7 days (**a**, **b** and **c** respectively) post injection of Matrigel and NIH3T3; left eye of rabbit 4 at 1, 3 and 7 days (**d**, **e** and **f** respectively) post injection of Matrigel only

**Fig. 2:**
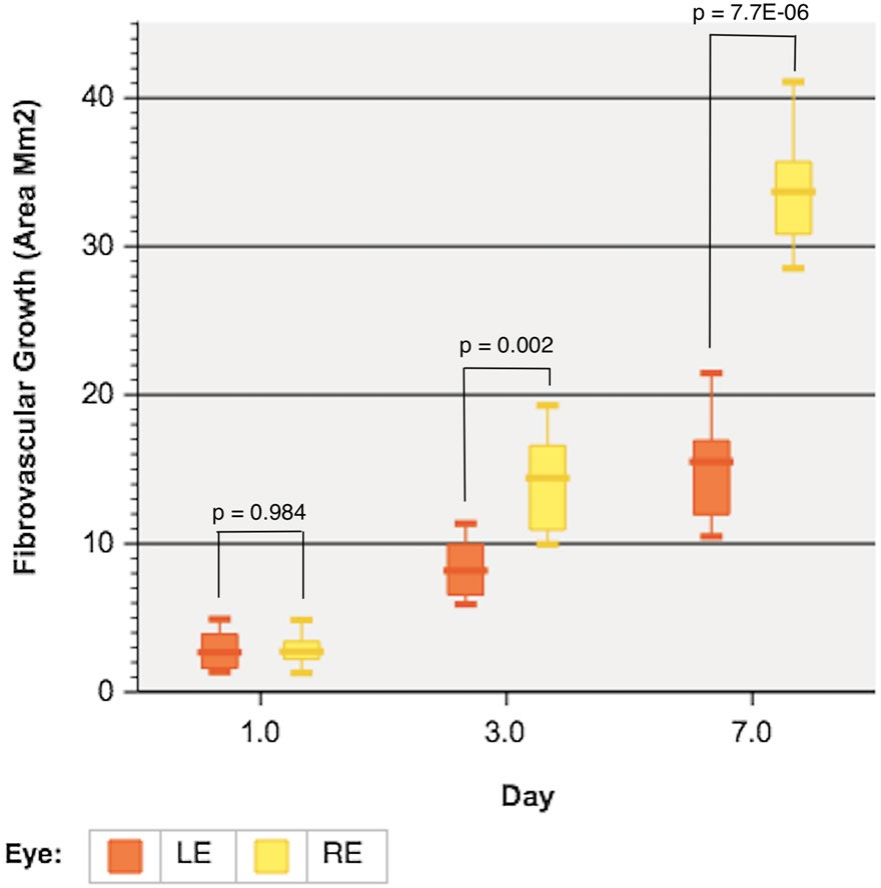
Analysis of the difference between the fibrovascular growth areas (mm^2^) observed at day 1, 3 and 7, post injection site in the animal model right (n = 6) and left eyes (n = 6) (6 rabbits, 12 eyes)

**Fig. 3:**
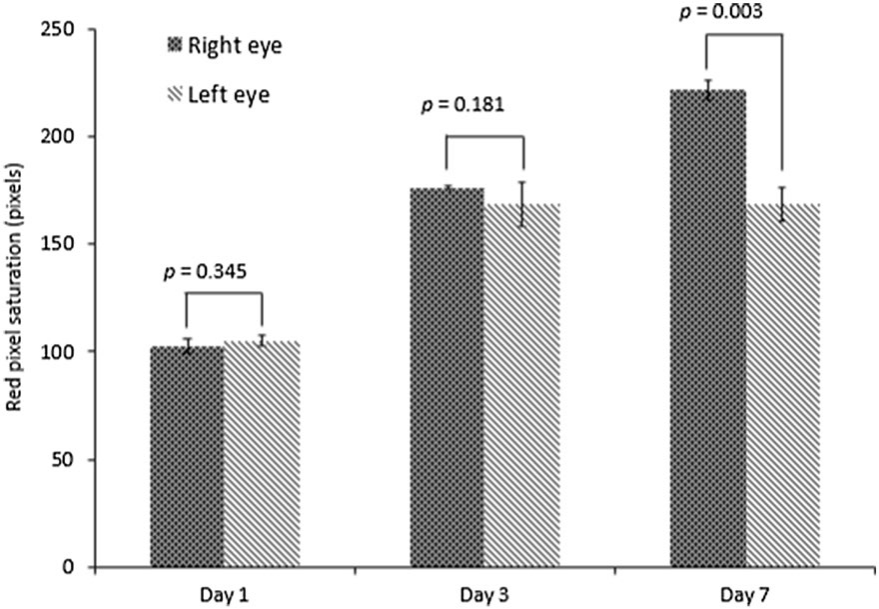
Red pixel saturation comparison analysis at day 1, 3 and 7, animal model right (n = 6) and left eyes (n = 6) (6 rabbits, 12 eyes)

All eyes (12 eyes) of the 6 rabbits developed conjunctival fibrovascular tissue after injection of Matrigel and NIH3T3 cells (n = 6, right eyes) or only Matrigel (n = 6, left eyes) at day 7 (Fig. 1). The mean area of fibrovascular tissue developed in the right eyes at day 1, 3 and 7 was 2.92 ± 1.21, 14.32 ± 3.47 and 33.97 ± 4.40 mm^2^ respectively. Left eyes had a mean fibrovascular tissue area of 2.91 ± 1.36, 8.41 ± 2.08 and 15.36 ± 3.89 mm^2^ at days 1, 3 and 7 respectively. The difference in fibrovascular growth areas observed between right and left eyes at day 3 and 7 was significant (p < 0.05) (Fig. 2). A significant increase in fibrovascular growth was also observed between days 1 and 3 (p = 0.001), days 1 and 7 (p < 0.001) and days 3 and 7 (p < 0.001) in right eyes. Likewise, a significant increase in fibrovascular growth was also observed between days 1 and 3 (p = 0.006), days 1 and 7 (p = 0.001) and days 3 and 7 (p = 0.001) in left eyes. No difference was seen between red pixel saturation of the fibrovascular tissue in right and left eyes at day 1 and 3. At day 7, right eyes exhibited significant (p < 0.05) higher red pixel saturation of the developed fibrovascular tissue, related to conjunctival vascularization, when compared with left eyes (Fig. 3).

**Fig. 4:**
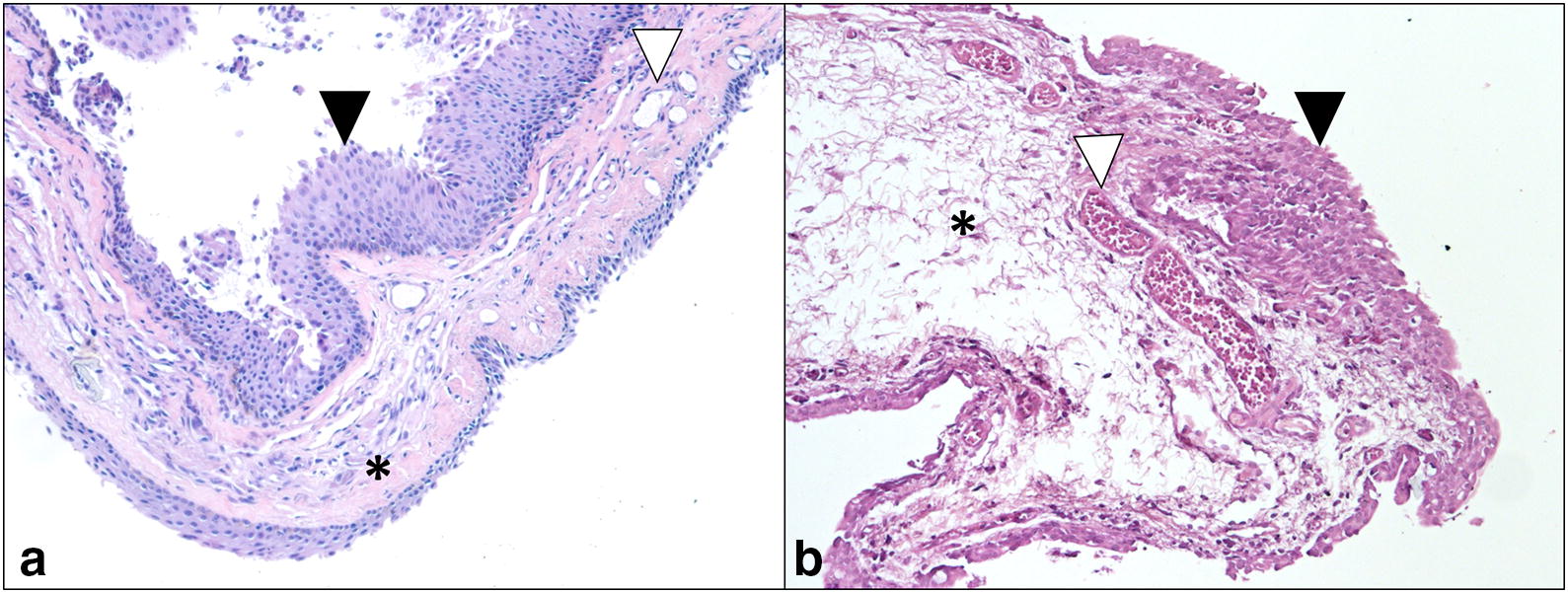
Comparison of the histopathological criteria evaluated in human pterygium **a** and the rabbit conjunctival fibrovascular tissue in a right eye **b**. Fibroconnective tissue (asterisk) is observed with a great amount of blood vessel (open triangle) coated by acanthotic squamous epithelium (filled triangle). In the rabbit tissue, edema interstitial is observed but not in human pterygium (H&E ×100)

Significant differences in the neovascularization pattern between rabbit induced pterygium tissue and human pterygium was observed in the histopathological analysis (Table 1). The average number of vessels found in the 6 analyzed lamellas of the right eyes was 29, while in the left eyes was 27; which is far from the 134 found in the human tissue. Five eyes of each treatment (right and left eyes) registered non- epithelial inflammation, resembling the changes observed in human pterygium. Stromal inflammation, absent in human pterygium, was observed in 1 of 6 right eyes and in 4 of the 6 left eyes. Acanthosis was observed in 4 right eyes and in 6 left eyes (Positive, grade 2 in human pterygium sample). Only one eye in each group (Matrigel + NIH 3T3 and Matrigel alone) showed elastosis in the same grade as human pterygium, while edema was observed in a similar grade between all right eyes specimens and human pterygium. Three left eyes showed grade 1 edema and three showed grade 2 edema. Figure 4 shows a representative image of the fibrovascular tissue developed in one rabbit (right eye, Matrigel + NIH 3T3) and the human pterygium.

**Table 1:** Comparison of the tissue characteristics of 13 lamellae, corresponding to the 6 right and 6 left eyes after 7 days of injection and a human pterygium

Eye	Rabbit 1		Rabbit 2		Rabbit 3		Rabbit 4		Rabbit 5		Rabbit 6		Human
	RE	LE	RE	LE	RE	LE	RE	LE	RE	LE	RE	LE	
Neovascularization	21	30	30	18	17	54	30	20	46	19	31	23	134
Epithelial inflammation	0	1	0	0	0	0	1	0	0	0	0	0	0
Stromal inflammation	1	1	1	0	0	0	1	0	1	0	1	1	0
Acanthosis	2	2	0	2	0	2	2	2	2	2	2	2	2
Elastosis	0	0	0	1	1	0	0	0	0	0	0	0	1
Edema	2	2	2	2	2	1	2	1	2	1	2	2	2

*RE* right eye, *LE* left eye

## Discussion

Pterygium is a frequent ophthalmological disease whose pathogenesis is not fully understood. The only definitive treatment available is surgical excision, with the disadvantage of a variable but persistent recurrence rate. Surgical alternatives and novel approaches involving autologous conjunctival grafts, extensive Tenon’s resection and amniotic membrane transplant in the excision area combined with the use of postsurgical antimetabolites to decrease the recurrence rate have shown promising results [5, 7]. However, the lack of knowledge about the subjacent molecular and cellular mechanisms have hindered the development of novel pharmacological treatments and therapeutic approaches. Consequently, the development of a pterygium animal model is an essential tool in the research for pathogenic events and novel surgical and pharmacological approaches.

Previous attempts in developing a rabbit models for pterygium were carried out by Kowk and Coroneo [3], who reported the use of incident ultraviolet (UV) scattered light at the temporal limbus to induce a predicted shaped and sized pterygium-like growth. Although this model reproduces the real UV light stimulation that is hypothesized to play a biologic key role in pterygium pathogenesis, the conjunctival/limbal stimulation with UV light required to produce significant conjunctival epithelial proliferation would be extensive, hence the need of a computational algorithm to simulate the predicted growth and final shape. Moreover, no histological analysis was reported to register the characteristics of the induced tissue growth.

In a different research, 6 week old athymic nude mice were used to induce pterygium with an injection of 1 × 10^4^ human epithelial pterygium cells followed by an injection of chondrocyte derived extracellular matrix 7, 10 or 14 days after the epithelial cell injection [11]. At day 17, a lesion growth was observed with higher significant size in the eyes injected with only epithelial cells than that observed in the eyes injected with cells and extracellular matrix. Their histological analysis revealed epithelial cells extending into the superficial stroma, neo-vessels, and extracellular matrix breakdown in the eyes injected with epithelial cells, but not in those injected with extracellular matrix. Similarly, Cox et al. used conjunctival injection with 1 × 10^4^ human epithelial pterygium cells mixed with 5 μL of Matrigel on nude athymic mice, observing a lesion growth on the injection site after 6 days [10]. They reported no lesion growth in mice injected with only Matrigel. In the histological analysis, neovascularization and migrating cells could be seen only in the epithelial cell injected mice conjunctivas.

Our model was conducted on young White New Zealand rabbits, an animal model that shares similarities with human ocular structure including size, thus providing an advantage over the surgical manipulation and the extent of pterygium-like tissue developed useful for further analysis. In our study, we compared the effect of the conjunctival injection of only Matrigel, in left eyes, and Matrigel with NIH3T3 fibroblasts, in right eyes. The concentration of injected cells and volume of Matrigel was the same used by Yang and Cox [10, 11]. Although following similar methods, Yang et al. [11] decided to wait until day 17 after injection to perform the histologic examination since it was only then when they observed a significant difference in the lesion size between their groups (eyes with only epithelial fibroblasts cells compared to the injection of extracellular matrix and cells). On the other hand, as evidenced on Fig. 2, we observed a significant difference in the lesion size since day 3 post injection between right eyes (NIH 3T3 cell + Matrigel) and left eyes (Matrigel). Hence, we decided to perform the histologic examination as early as day 7 post injection. However, relevant data and observations can emerge from future protocols assessing the histological characteristics between the lesions in different post injection times.

Although both Matrigel and chondrocyte derived extracellular matrix are used to promote cell proliferation, the former is known for its gel composition at room temperature containing bFGF, IGF, EGF, laminin, collagen 1, and heparin sulfate proteoglycan [12], which exerts a slow release characteristic that favors angiogenesis [13–16]. In the mice model injected with Matrigel reported by Cox, there was no tissue growth at day 6. Our model suggests that the combination of NIH3T3 cells (an immortalized mouse fibroblast line) with Matrigel, enhances the growth of the pterygium-like tissue. This was confirmed by the macroscopic clinical analysis performed to assess red-pixel saturation, which can correlate to hyperemia, neovascularization and other histological events; and tissue growth area measured in square millimeters.

A histological analysis using a scale of 6 parameters commonly observed in human pterygium [17–20] was carried out. The most distinctive feature of human pterygium is neovascularization [19, 21]. In our analysis, tissues of both groups showed neovascularization with a slight increased level in right eyes, but in a lower grade than that observed in human pterygium [20]. Nonetheless, the number of new vessels in human pterygium has been reported as extremely variable [19, 20]. In human pterygium, redness and fleshiness are correlated with the vascular density and stromal fibrosis, while the extent of the pterygium over the cornea has an inverse relation with the stromal elastosis [18]. This is in accordance with our results, in which higher redness and vascularization was observed in right eyes. No invasion over the cornea was observed after 7 days, further experiments analyzing the growth of the tissue over a longer period will provide information about the correlation of this parameter and the stromal elastosis level observed.

No epithelial inflammation was seen in the tissue induced in right or left eyes, paralleling the histopathology of primary human pterygium. Elastosis was seen only in one eye of each treatment group. The latter parameter is highly associated to chronic UV radiation exposure [17], and it is reported as a key histological characteristic in primary pterygium and minimal in recurrent pterygium [20]. The absence of significant elastotic changes in the analyzed tissues could be associated with the method used to induce the pterygium-like tissue formation (eliciting inflammatory immune response and/or fibroblastic cell proliferation, not associated with UV light exposure) and to the short period after which histological analysis was performed. Otherwise, cellular edema was similar between right eyes and human pterygium, comparable with the observations of previous reports [19]. Although similar protocols in mice models [10, 11] have succeeded in eliciting and inflammatory response and tissue growth over the injection site (subconjunctival), histologic descriptions when available, have only shown neo-vessels, cell migration and extracellular matrix breakdown with no evidence of acanthosis, elastosis or stromal/epithelial inflammation.

## Conclusions

In conclusion, Matrigel alone or in combination with NIH3T3 cells injected into the rabbit conjunctiva can promote tissue growth with clinical and histological characteristics like those of human pterygium, including neovascularization, acanthosis, stromal inflammation, and edema. The combination of Matrigel with NIH3T3 cells seems to have an additive effect on the size and redness of the pterygium-like tissue developed. The presented animal model combines successfully the formation of vascular conjunctival lesion with the convenient size of the rabbit eye, making it suitable for its implementation on both basic pharmacologic/molecular research on pterygium pathogenesis and on novel surgical techniques.
